# Effect of olmesartan and amlodipine on serum angiotensin-(1–7) levels and kidney and vascular function in patients with type 2 diabetes and hypertension

**DOI:** 10.1186/s13098-023-00987-1

**Published:** 2023-03-11

**Authors:** Kyuho Kim, Ji Hye Moon, Chang Ho Ahn, Soo Lim

**Affiliations:** 1 Department of Internal Medicine, College of Medicine, The Catholic University of Korea, Seoul, South Korea; 2 Department of Internal Medicine, Seoul National University College of Medicine and Seoul National University Bundang Hospital, 300 Gumi-dong, Bundang-gu, Seongnam, 463-707 South Korea

**Keywords:** Angiotensin-converting enzyme 2, Angiotensin-(1–7), Olmesartan, Amlodipine, Albuminuria

## Abstract

**Background:**

Recent studies suggest that angiotensin-converting enzyme 2 (ACE2) and angiotensin-(1–7) [Ang-(1–7)] might have beneficial effects on the cardiovascular system. We investigated the effects of olmesartan on the changes in serum ACE2 and Ang-(1–7) levels as well as kidney and vascular function in patients with type 2 diabetes and hypertension.

**Methods:**

This was a prospective, randomized, active comparator-controlled trial. Eighty participants with type 2 diabetes and hypertension were randomized to receive 20 mg of olmesartan (N = 40) or 5 mg of amlodipine (N = 40) once daily. The primary endpoint was changes of serum Ang-(1–7) from baseline to week 24.

**Results:**

Both olmesartan and amlodipine treatment for 24 weeks decreased systolic and diastolic blood pressures significantly by > 18 mmHg and > 8 mmHg, respectively. Serum Ang-(1–7) levels were more significantly increased by olmesartan treatment (25.8 ± 34.5 pg/mL → 46.2 ± 59.4 pg/mL) than by amlodipine treatment (29.2 ± 38.9 pg/mL → 31.7 ± 26.0 pg/mL), resulting in significant between-group differences (P = 0.01). Serum ACE2 levels showed a similar pattern (6.31 ± 0.42 ng/mL → 6.74 ± 0.39 ng/mL by olmesartan treatment vs. 6.43 ± 0.23 ng/mL → 6.61 ± 0.42 ng/mL by amlodipine treatment; P < 0.05). The reduction in albuminuria was significantly associated with the increases in ACE2 and Ang-(1–7) levels (*r* =  − 0.252 and *r* =  − 0.299, respectively). The change in Ang-(1–7) levels was positively associated with improved microvascular function (*r* = 0.241, P < 0.05). Multivariate regression analyses showed that increases in serum Ang-(1–7) levels were an independent predictor of a reduction in albuminuria.

**Conclusions:**

These findings suggest that the beneficial effects of olmesartan on albuminuria may be mediated by increased ACE2 and Ang-(1–7) levels. These novel biomarkers may be therapeutic targets for the prevention and treatment of diabetic kidney disease.

*Trial registration:* ClinicalTrials.gov NCT05189015.

**Supplementary Information:**

The online version contains supplementary material available at 10.1186/s13098-023-00987-1.

## Introduction

The renin-angiotensin system (RAS) plays important roles in the regulation of normal physiology and the pathogenesis of cardiovascular diseases (CVDs), including atherosclerosis, hypertension, myocardial infarction, and cardiac remodeling [1, 2]. The well-known components of “classical” RAS include angiotensinogen, angiotensin I, angiotensin II (AngII), renin, and angiotensin-converting enzyme (ACE). Among these, AngII, which is a major effector molecule, exerts its biological actions via the AngII type 1 receptor, contributing to the development of CVD [3].

There are “nonclassical” RAS pathways, which include ACE2, its product angiotensin-(1–7) [Ang-(1–7)], and Mas receptor [4]. These components are thought to have protective effects on CVDs, although the exact mechanisms underlying such effects are not completely understood [5]. In rodent models, ACE2, through catabolism of AngII [6–8] and Ang-(1–7) [9–12], showed beneficial effects on blood pressure (BP), atherosclerosis, cardiac remodeling, and heart failure. A few animal model studies suggested that ACE inhibitor or AngII receptor blocker (ARB) treatments can cause ACE2 upregulation with consequential beneficial effects on CVDs [13]. More specifically, a mice study showed that treatment with olmesartan, which is an ARB, inhibits cardiac hypertrophy independently of BP via AngII type 1 receptor blockade and partly through the enhancement of ACE2/Ang-(1–7)/Mas axis pathway [14]. A human study also showed that among many antihypertensive drugs, only olmesartan treatment led to significantly higher urinary ACE2 levels than those in the control group [15]. In that study, olmesartan was an independent predictor of urinary ACE2 levels, with potential additional renoprotective effects [15]. However, the study was an observational study with a small sample size, and it did not measure the plasma levels of ACE2 or Ang-(1–7).

Considering that ACE inhibitors or ARBs are the first-line drugs for antihypertensive treatment in patients with type 2 diabetes (T2D) and have potential “nonclassical” RAS-mediated benefits, it is meaningful to measure the change in the plasma levels of “nonclassical” RAS components after drug treatment. This could help clinicians select the more appropriate drugs between ACE inhibitors and ARBs with solid evidence.

Recently, ACE2 has received much attention because it can serve as an entry receptor for severe acute respiratory syndrome coronavirus 2 [16]. ACE2 is widely expressed in humans, including in the myocardium, vasculature, pancreas, kidneys, intestines, and lungs [17]. Alteration of ACE2 levels by RAS blocking agents can be associated with coronavirus disease 2019 (COVID-19) occurrence and its severity.

In the present study, we aimed to investigate the changes in serum ACE2 levels, Ang-(1–7) levels, and their association with kidney and vascular function after using olmesartan, compared with a conventional antihypertensive drug (amlodipine) in patients with T2D and hypertension.

## Methods

### Study design

This was a 24-week, prospective, randomized, active comparator-controlled trial conducted at the Seoul National University Bundang Hospital. After 1:1 randomization, patients received 20 mg of olmesartan or 5 mg of amlodipine once daily. After 12 weeks, the dose was titrated to 40 mg of olmesartan or 10 mg of amlodipine once daily for the next 12 weeks for the participants with systolic blood pressure (SBP) > 160 mmHg or diastolic blood pressure (DBP) > 100 mmHg. During this study period, medications except for olmesartan and amlodipine were not changed or added. The study was registered at ClinicalTrials.gov (NCT05189015).

### Study participants

The inclusion criteria were age ≥ 30 years; T2D and glycated hemoglobin (HbA1c) levels = 6.5–10.0%; SBP = 140–180 mmHg or DBP = 85–110 mmHg, considering a target BP of < 140/85 mmHg for patients with T2D according to the guidelines of Korean Diabetes Association [18], to exclude patients with severe hypertension [19]; and no change in the dose of statins in the previous 3 months. The exclusion criteria were a history of taking RAS inhibitors (ACE inhibitors or ARBs) or calcium channel blockers (CCBs) in the previous 3 months, pregnancy, lactation, confirmed CVDs within 3 months of screening, active liver disease (aspartate transaminase/alanine transaminase levels > threefold of the upper limit of normal), hyperkalemia (serum potassium levels > 5.0 mEq/L), and any previous cancer within 5 years. Patients with serum creatinine levels > 2.0 mg/dL (advanced chronic kidney disease) were also excluded for their safety. Participants meeting all inclusion and none of the exclusion criteria were randomized to the study. Randomization was conducted sequentially as the participants became eligible.

### Study objectives

The primary objective of this study was to measure changes in serum Ang-(1–7) from baseline to week 24. The key secondary objectives were to measure the following: (1) changes in BP from baseline to week 24; (2) changes in serum ACE2 levels, plasma renin activity (PRA), and aldosterone from baseline to week 24; (3) changes in the ratio of urinary protein-to-creatinine concentration (UPCR, mg/g) or urinary albumin-to-creatinine concentration (UACR, mg/g); (4) changes in flow-mediated vasodilatation (FMD) and microcirculation from baseline to week 24; (5) changes in body mass index and body fat percentile from baseline to week 24; and (6) changes in glucose metabolism parameters (HbA1c, fasting plasma glucose [FPG], and insulin), lipid profiles (total cholesterol, triglycerides, high-density lipoprotein [HDL] cholesterol, and low-density lipoprotein [LDL] cholesterol), and high-sensitivity C-reactive protein (hsCRP) from baseline to week 24.

### Anthropometric parameters

Height was measured while wearing no shoes (in cm). Weight was measured while wearing light clothes and no shoes (in kg). Waist circumference (in cm) was measured midway between the lowest rib and the iliac crest, in the morning before having breakfast. Body mass index was calculated as weight (in kg) divided by the square of the height (in meters). Body composition was estimated using a multifrequency whole-body bioelectrical impedance analysis (InBody 720, InBody Co., Seoul, South Korea).

### Measurement of blood pressure and pulse

BP and heart rate were measured in a seated position with the arms raised to the level of the heart and in a supported position. One pulse measurement was taken after the participant had been sitting and resting for at least 5 min and before blood samples were taken. BP was measured using a standardized cuff adapted to the size of the participant’s arm.

### Laboratory assessments

Blood samples for laboratory measurement were collected after fasting for at least 8 h. Fasting plasma levels of glucose, total cholesterol, triglycerides, HDL-cholesterol, LDL-cholesterol, and serum creatinine were measured using standard automated laboratory methods (Hitachi 747; Hitachi, Tokyo, Japan). The estimated glomerular filtration rate (eGFR) was calculated using the creatinine-based Chronic Kidney Disease Epidemiology Collaboration equation. HbA1c levels were measured using a high-performance liquid chromatography Variant II Turbo analyzer (Bio-Rad Laboratories, Hercules, CA, USA) in the National Glycohemoglobin Standardization Program level II certified laboratory at the Seoul National University Bundang Hospital. Plasma C-peptide and insulin levels were measured using a radioimmunoassay (RIA; Linco, St. Louis, MO, USA). Serum aspartate transaminase and alanine transaminase levels were measured using an autoanalyzer (TBA-200FR, Toshiba, Tokyo, Japan). Serum hsCRP levels were measured using an automated latex turbidimetric immunoassay method (CRP Latex X2, Denka Seiken, Tokyo, Japan). Urinary protein or albumin levels were measured using a turbidimetric assay (A&T 502X, A&T, Tokyo, Japan). Urinary creatinine levels were measured using the Jaffe method (Hitachi 7170, Hitachi, Tokyo, Japan). Proteinuria and albuminuria were assessed based on UPCR and UACR, respectively.

Insulin resistance (IR) index and pancreatic β-cell function (β) assessed from the homeostasis model assessment (HOMA) were calculated using the following formula: HOMA-IR = fasting plasma insulin (μU/mL) × FPG (mg/dL)/405; HOMA-β = 360 × fasting plasma insulin (μU/mL)/[FPG (mg/dL) − 63] [20].

PRA was measured using a PRA RIA kit (TFB Inc., Tokyo, Japan), and plasma aldosterone levels were measured using the SPAC-S aldosterone RIA kit (TFB Inc.). Serum levels of Ang-(1–7) and ACE2 were measured using ELISA kits (Human Angiotensin (1–7) ELISA kit Cat. No. MBS084052 and Human ACE2 ELISA kit Cat. No. MBS824839, respectively; MyBioSource, San Diego, CA, USA) according to the manufacturer’s instructions [21].

### Vascular function assessment

#### Flow-mediated vasodilation

Endothelial-dependent FMD was measured using high-resolution ultrasonography according to the guidelines [22]. After supine rest for at least 5 min, a baseline rest image of the brachial artery was acquired. Then, the cuff was inflated to at least 50 mmHg higher than the SBP of the upper arm for 2 min. The longitudinal image of the artery was recorded continuously from 30 s before to 2 min after cuff deflation. The diameter of the artery was measured from one media-adventitia interface to the other. FMD change (%) was defined using the change in artery diameter between baseline and 1 min after cuff deflation.

#### Microcirculation

To assess microvascular function, postocclusive reactive hyperemia (PORH) was measured using the PeriFlux 400 laser Doppler (Perimed, Stockholm, Sweden) [23]. The laser Doppler probe was applied at the dorsum of the foot between the first and second metatarsal bones. After the patients had rested for 10 min, a 4-min occlusion of the lower limb was performed using a cuff placed on the ankle. The pressure of the cuff was at least 50 mmHg above the SBP of the ankle. Then, the flow within 1 min after cuff deflation was recorded. PORH was expressed as arbitrary perfusion units, and PORH change (%) was defined using the change in PORH between baseline and 1 min after cuff deflation.

### Safety assessments

Safety assessments were performed throughout the study and included regular monitoring of physical examination, pregnancy evaluation, and clinical laboratory data, including an electrocardiogram. All and serious adverse events were recorded for all participants.

### Statistical analysis

No previous interventional studies investigating the effect of amlodipine or olmesartan on Ang-(1–7) levels exist. Therefore, we used the changes in ACE2 levels instead of Ang-(1–7) levels in the sample size calculation. In a previous study, urinary ACE2 levels were significantly increased by > 50% after olmesartan treatment [24]. We assumed a 40% increase in Ang-(1–7) levels after olmesartan treatment in a conservative manner. Moreover, no study investigating the effect of amlodipine on Ang-(1–7) or ACE2 levels exists. Considering that amlodipine is a CCB, no change in Ang-(1–7) or ACE2 levels after amlodipine treatment is expected. For this study, we assumed a 10% increase in Ang-(1–7) levels after amlodipine treatment in a conservative manner. Based on this criterion, the sample size was calculated with the assumption of a 30% intergroup difference in the changes in serum ACE2 levels from baseline to week 24 with a standard deviation (SD) of 15%, yielding 34 patients per group for a 90% statistical power with α = 0.05. Assuming a 15% dropout rate, a minimal sample size of 40 patients per group (1:1 randomization) was estimated to be required. Eligible participants at screening who met the inclusion criteria were randomly assigned into either the olmesartan or the amlodipine group aiming for an equal number of participants per treatment group. The randomization scheme in blocks was generated using IBM SPSS software, version 25.0 (IBM Corp., Armonk, NY, USA). Per-protocol analyses were performed except analyses of baseline characteristics and safety assessment of study participants.

Data were expressed as mean ± SD, median (25–75th percentile), or number (%) as indicated. Comparisons of continuous variables were performed using the two-sample *t* test (or Wilcoxon rank sum test) or paired *t* test (or Wilcoxon signed-rank test). Comparisons of categorical variables were performed using the chi-square test (or Fisher’s exact test) or McNemar’s test. Spearman’s and Pearson’s correlation analyses were used to evaluate the correlation between variables. Logarithmically transformed values of triglyceride, HOMA-IR, UACR, UPCR, hsCRP, Ang-(1–7), PRA, and PORH were used for statistical analysis. Using established risk factors for proteinuria or albuminuria and variables of interest, univariate regression analyses and stepwise multivariate regression analyses were performed to identify independent determinants for the changes in UPCR and UACR. P < 0.05 was considered statistically significant. Statistical analyses were performed using the IBM SPSS software, version 25.0 (Armonk, NY).

## Results

**Fig. 1:**
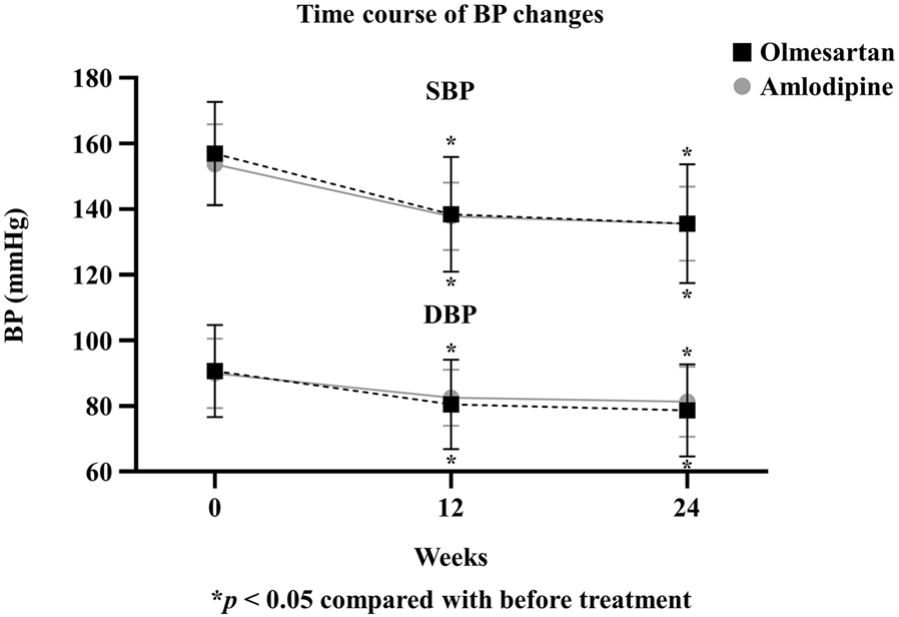
Changes in blood pressure 12 and 24 weeks after treatment with olmesartan (N = 36) or amlodipine (N = 35). *BP* blood pressure

A total of 80 patients were randomized to the olmesartan (N = 40) or amlodipine (N = 40) group. Of these, 71 patients (88.5%) completed 24 weeks: 36 in the olmesartan group and 35 in the amlodipine group (Additional file 1: Fig. S1). No differences in baseline clinical and biochemical characteristics were observed between the two groups (Table 1). The number of men was 23 and 30 in the olmesartan and amlodipine groups, respectively (P > 0.05). At the baseline, SBP was > 150 mmHg and DBP was > 85 mmHg in both groups. After 24 weeks of treatment, SBP decreased by 21.3 mmHg and 18.0 mmHg in the olmesartan and amlodipine groups, respectively, leading to no between-group difference in the extent of SBP change. Similarly, DBP decreased by 12.1 mmHg and 8.7 mmHg in the olmesartan and amlodipine groups, respectively, leading to no between-group difference in the extent of DBP change (Fig. 1 and Table 2). No difference was observed in the proportion of maximum dose in olmesartan (36.1%) or amlodipine (28.6%) groups (P > 0.05).

**Table 1:** Baseline characteristics of the study participants

	Olmesartan (N = 40)	Amlodipine (N = 40)	P
Age, year	56.5 ± 14.9	56.3 ± 12.5	NS
Body mass index, kg/m^2^	26.0 ± 3.7	26.3 ± 3.5	NS
Body fat percentile, %	30.2 ± 5.9	29.4 ± 6.3	NS
Waist circumference, cm	91.4 ± 8.9	92.5 ± 8.6	NS
Systolic blood pressure, mmHg	156.1 ± 15.4	154.2 ± 12.6	NS
Diastolic blood pressure, mmHg	89.7 ± 13.8	89.7 ± 10.4	NS
Heart rate, beats/min	85.0 ± 12.7	85.3 ± 12.6	NS
Fasting glucose, mg/dL	146.8 ± 47.5	161.0 ± 41.5	NS
Postprandial 2 h glucose, mg/dL	250.5 ± 65.9	237.9 ± 61.5	NS
HbA1c, %	7.4 ± 1.4	7.5 ± 1.3	NS
C-peptide, ng/mL	2.6 ± 1.2	2.7 ± 1.2	NS
Insulin, μIU/mL	9.1 ± 5.1	9.8 ± 5.3	NS
Total cholesterol, mg/dL	182.3 ± 44.3	178.8 ± 49.3	NS
Triglyceride^a^, mg/dL	119.0 (97.3–265.3)	166.5 (113.3–223.0)	NS
HDL-cholesterol, mg/dL	50.0 ± 10.5	51.8 ± 13.0	NS
LDL-cholesterol, mg/dL	111.0 ± 30.0	106.2 ± 34.5	NS
AST, IU/L	31.6 ± 14.2	33.9 ± 18.5	NS
ALT, IU/L	24.9 ± 21.8	38.5 ± 23.5	NS
eGFR, ml/min/1.73 m^2^	94.3 ± 25.9	99.0 ± 19.6	NS
hsCRP^a^, mg/dL	0.06 (0.03–0.18)	0.06 (0.04–0.12)	NS
UACR^a^, mg/g	29.5 (11.1–84.4)	28.3 (11.0–148.0)	NS
UPCR^a^, mg/g	109.8 (77.2–249.9)	120.5 (86.8–275.8)	NS
Medication			
Metformin, N (%)	36 (94.7)	37 (92.5)	NS
Sulfonylurea, N (%)	15 (39.5)	15 (37.5)	NS
DPP-4 inhibitor, N (%)	15 (39.5)	14 (35.0)	NS
SGLT-2 inhibitor, N (%)	3 (7.9)	6 (15.0)	NS
GLP-1 receptor agonist, N (%)	0 (0.0)	1 (2.5)	NS
Thiazolidinedione, N (%)	3 (7.9)	2 (5.0)	NS

Data are expressed as mean ± standard deviation (SD), median (25–75th percentile), or number (%). ^a^P value obtained after log transformation of the data. *eGFR* estimated glomerular filtration rate; *UACR* urinary albumin-to-creatinine ratio; *UPCR* urinary protein-to-creatinine ratio, *DPP-4* dipeptidyl peptidase-4, *SGLT-2* sodium-glucose cotransporter-2, *GLP-1* glucagon like peptide-1, *NS* not significant

**Table 2:** Changes in biochemical parameters after treatment from baseline to week 24

	Olmesartan (N = 36)				Amlodipine (N = 35)				
	Baseline	Week 24	Change	*P	Baseline	Week 24	Change	*P	^†^P
Systolic BP, mmHg	156.9 ± 15.8	135.6 ± 18.2	− 21.3 ± 19.5	< 0.001	153.6 ± 12.2	135.6 ± 11.3	− 18.0 ± 11.5	< 0.001	0.395
Diastolic BP, mmHg	90.7 ± 14.0	78.6 ± 14.1	− 12.0 ± 14.0	< 0.001	90.0 ± 10.6	81.3 ± 10.6	− 8.7 ± 11.3	< 0.001	0.273
Heart rate, beats/min	84.9 ± 11.9	86.7 ± 14.4	1.8 ± 10.5	0.325	85.3 ± 13.3	84.1 ± 12.7	− 1.3 ± 8.5	0.390	0.191
Body mass index, kg/m^2^	26.1 ± 3.6	26.1 ± 3.6	− 0.1 ± 1.0	0.621	26.7 ± 3.5	26.5 ± 3.4	− 0.2 ± 0.6	0.073	0.625
Body fat percentile, %	30.0 ± 5.5	29.6 ± 5.2	− 0.4 ± 2.1	0.233	28.9 ± 5.3	28.9 ± 5.4	− 0.1 ± 1.5	0.845	0.392
Total cholesterol, mg/dL	182.4 ± 42.6	174.5 ± 44.7	− 7.9 ± 33.7	0.171	173.3 ± 47.5	169.3 ± 37.0	− 4.0 ± 30.6	0.445	0.615
Triglyceride^a^, mg/dL	119.0 (97.3–207.0)	132.5 (74.5–261.3)	8.0 (–37.8–58.8)	0.845	164.0 (109.0–233.0)	127.0 (110.0–206.0)	− 11.0 (− 59.0–37.0)	0.170	0.245
HDL-cholesterol, mg/dL	50.6 ± 10.7	49.7 ± 11.2	− 0.9 ± 6.9	0.416	51.4 ± 13.6	51.3 ± 14.7	− 0.1 ± 9.2	0.956	0.657
LDL-cholesterol, mg/dL	111.3 ± 30.0	101.9 ± 33.1	− 9.4 ± 22.4	0.016	101.8 ± 32.1	100.4 ± 31.6	− 1.4 ± 22.9	0.725	0.138
Fasting glucose, mg/dL	144.9 ± 41.2	142.5 ± 40.7	− 2.4 ± 45.1	0.749	159.0 ± 42.3	168.6 ± 54.2	9.6 ± 48.4	0.249	0.282
PP2, mg/dL	253.9 ± 62.4	243.1 ± 74.0	− 10.8 ± 90.7	0.478	235.8 ± 63.3	265.0 ± 83.2	29.2 ± 81.3	0.041	0.055
HbA1c, %	7.3 ± 1.2	7.4 ± 1.3	0.1 ± 0.7	0.437	7.2 ± 0.7	7.6 ± 1.3	0.3 ± 1.1	0.075	0.279
Insulin, μIU/mL	8.9 ± 5.2	9.6 ± 4.5	0.7 ± 3.9	0.273	9.9 ± 5.3	9.9 ± 5.3	0.0 ± 2.2	0.988	0.341
HOMA-IR^a^	2.6 (1.9–5.0)	3.1 (1.8–4.2)	0.4 (–1.5–0.7)	0.189	3.2 (2.7–4.3)	3.4 (2.5–5.1)	0.2 (–1.0–0.7)	0.394	0.420
HOMA-β	49.2 ± 40.8	53.8 ± 39.2	4.6 ± 39.9	0.497	52.3 ± 59.8	45.1 ± 43.7	− 7.2 ± 34.3	0.223	0.188
AST, IU/L	32.0 ± 14.8	29.5 ± 12.0	− 2.5 ± 10.8	0.174	35.0 ± 19.1	34.1 ± 17.7	− 0.9 ± 15.0	0.738	0.598
ALT, IU/L	35.4 ± 22.9	35.2 ± 24.3	− 0.2 ± 18.0	0.941	41.4 ± 23.8	48.5 ± 32.1	7.1 ± 21.3	0.056	0.121
Na, mEq/L	140.3 ± 1.8	139.8 ± 2.0	− 0.5 ± 1.9	0.113	140.3 ± 1.9	140.4 ± 2.0	0.1 ± 1.7	0.761	0.158
K, mEq/L	4.3 ± 0.4	4.5 ± 0.4	0.2 ± 0.4	0.008	4.5 ± 0.3	4.5 ± 0.3	0.1 ± 0.3	0.241	0.005
Cl, mEq/L	102.8 ± 2.6	102.7 ± 2.7	− 0.1 ± 2.6	0.748	102.8 ± 2.0	102.4 ± 2.1	-0.4 ± 1.9	0.198	0.593
eGFR, ml/min/1.73m^2^	94.4 ± 22.5	95.6 ± 27.1	1.2 ± 16.8	0.671	100.5 ± 18.0	101.5 ± 17.5	1.0 ± 17.6	0.738	0.962
UACR^a^, mg/g	29.5 (10.2–84.4)	19.6 (8.9–44.0)	− 3.9 (− 37.1–2.1)	0.005	35.0 (11.1–157.4)	26.7 (10.0–206.4)	–1.6 (–4.3–22.6)	0.155	0.002
UPCR^a^, mg/g	109.8 (76.9–249.9)	113.5 (67.7–152.2)	3.0 (− 54.0–38.4)	0.249	120.6 (93.4–292.3)	133.9 (88.3–353.1)	4.8 (–3.64–14.4)	0.142	0.047
hsCRP^a^, mg/dL	0.05 (0.03–0.13)	0.06 (0.03–0.11)	0.00 (–0.02–0.02)	0.621	0.06 (0.04–0.12)	0.06 (0.04–0.12)	0.00 (–0.02–0.01)	0.473	0.436
Renin-angiotensin system									
Ang-(1–7)^a^, pg/mL	14.7 (5.1–30.8)	30.3 (15.2–51.7)	10.7 (0.2–22.3)	< 0.001	18.8 (13.4–29.6)	26.7 (13.7–38.1)	3.3 (− 4.3–16.3)	0.212	0.010
ACE2, ng/mL	6.31 ± 0.42	6.74 ± 0.39	0.43 ± 0.32	< 0.001	6.43 ± 0.23	6.61 ± 0.42	0.18 ± 0.34	0.003	0.002
PRA^a^, ng/ml/hr	0.8 (0.5–1.7)	4.2 (1.6–8.7)	2.7 (0.3–6.8)	< 0.001	1.2 (0.6–1.9)	1.4 (0.9–2.5)	0.6 (–0.4–0.8)	0.089	< 0.001
Aldosterone, ng/dL	18.0 ± 7.8	13.5 ± 7.2	− 4.5 ± 7.7	0.001	17.9 ± 6.0	19.0 ± 8.7	1.0 ± 7.0	0.388	0.002
Vascular function									
FMD baseline (mm)	4.8 ± 0.6	4.7 ± 0.6	− 0.1 ± 0.4	0.485	5.0 ± 0.7	5.0 ± 0.7	–0.0 ± 0.4	0.547	0.897
FMD after 1 min (mm)	5.2 ± 0.6	5.2 ± 0.7	− 0.1 ± 0.5	0.744	5.4 ± 0.7	5.4 ± 0.7	–0.1 ± 0.4	0.224	0.919
FMD change (%)	9.7 ± 5.2	10.3 ± 7.5	0.6 ± 5.7	0.519	9.2 ± 4.2	8.5 ± 4.1	–0.7 ± 5.5	0.442	0.317
PORH baseline (PU)	17.4 ± 7.5	19.5 ± 9.4	2.1 ± 10.4	0.234	17.4 ± 7.4	19.6 ± 7.7	2.2 ± 10.7	0.240	0.981
PORH after 1 min (PU)	56.7 ± 20.2	61.4 ± 31.9	4.6 ± 28.3	0.332	62.1 ± 21.4	58.8 ± 33.9	–3.3 ± 35.2	0.583	0.298
PORH change^a^ (%)	223.2 (107.1–366.8)	191.9 (122.1–343.6)	–19.5 (–205.1–92.5)	0.726	261.3 (175.8–359.7)	177.5 (105.3–253.4)	–75.2 (–146.3–46.3)	0.026	0.189

Data are expressed as mean ± standard deviation (SD) or median (25–75th percentile). ^a^Log-transformed values were used for comparison. *P values were calculated using a paired *t* test between the values at the baseline and after treatment. ^†^P values were calculated using Student’s *t* test for delta changes between the two groups

PP2, postprandial 2-h glucose; HOMA-IR, homeostasis model assessment of insulin resistance; HOMA-β, homeostasis model assessment of β-cell function; UACR, urinary albumin-to-creatinine ratio; UPCR, urinary protein-to-creatinine ratio; hsCRP, high-sensitivity C-reactive protein; PRA, plasma renin activity; ACE2, angiotensin-converting enzyme-2; FMD, flow-mediated vasodilation; PORH, postocclusive reactive hyperemia; PU, perfusion unit

**Fig. 2:**
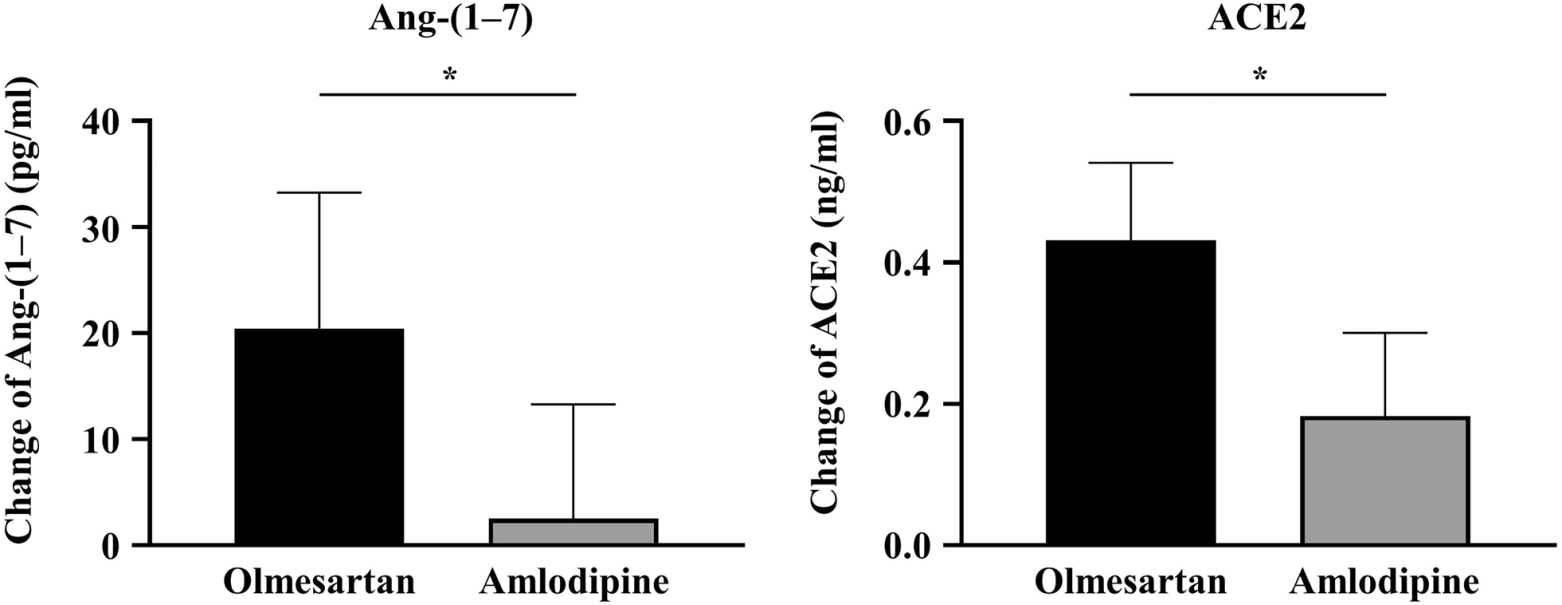
Changes in serum Ang-(1–7) and ACE2 levels 24 weeks after treatment with olmesartan (N = 36) or amlodipine (N = 35). *ACE2* angiotensin-converting enzyme 2; Ang-(1–7), angiotensin-(1–7)

Notably, Ang-(1–7) levels, which were the primary endpoint of this study, increased significantly by olmesartan treatment but they did not change much by amlodipine treatment. Thus, the extent of changes was significantly different between the groups. Similarly, serum ACE2 levels increased significantly in both groups, but the increase was greater in the olmesartan group than in the amlodipine group, leading to a significant difference between the groups. In the olmesartan group, PRA increased significantly but aldosterone levels decreased significantly. These changes were not observed in the amlodipine group (Fig. 2 and Table 2).

In the assessment of FMD for vascular function, the FMDs at baseline and after 1-min stimulation did not change after 24-week olmesartan or amlodipine treatment. In the PORH measurement for the assessment of microvascular circulation, the baseline value increased in both groups. However, the stimulated value of PORH after 1 min of stimulation was greater in the olmesartan group than in the amlodipine group, leading to a significant decrease in PORH change (%) by amlodipine treatment but not by olmesartan treatment. Nevertheless, no difference was observed in the extent of change in PORH between groups (Table 2).

No differences were observed for body mass index, body fat percentile, fasting glucose, HbA1c, insulin, HOMA-IR, HOMA-β, lipid profiles, and hsCRP between the groups. However, 2-h postprandial glucose level was significantly increased in the amlodipine group while it was maintained in the olmesartan group, resulting in a borderline significant difference between the groups (P = 0.055). In addition, urinary albumin and protein excretion rates showed a tendency to decrease in the olmesartan group whereas they were not changed in the amlodipine group, resulting in a significant difference between the groups (Table 2).

**Fig. 3:**
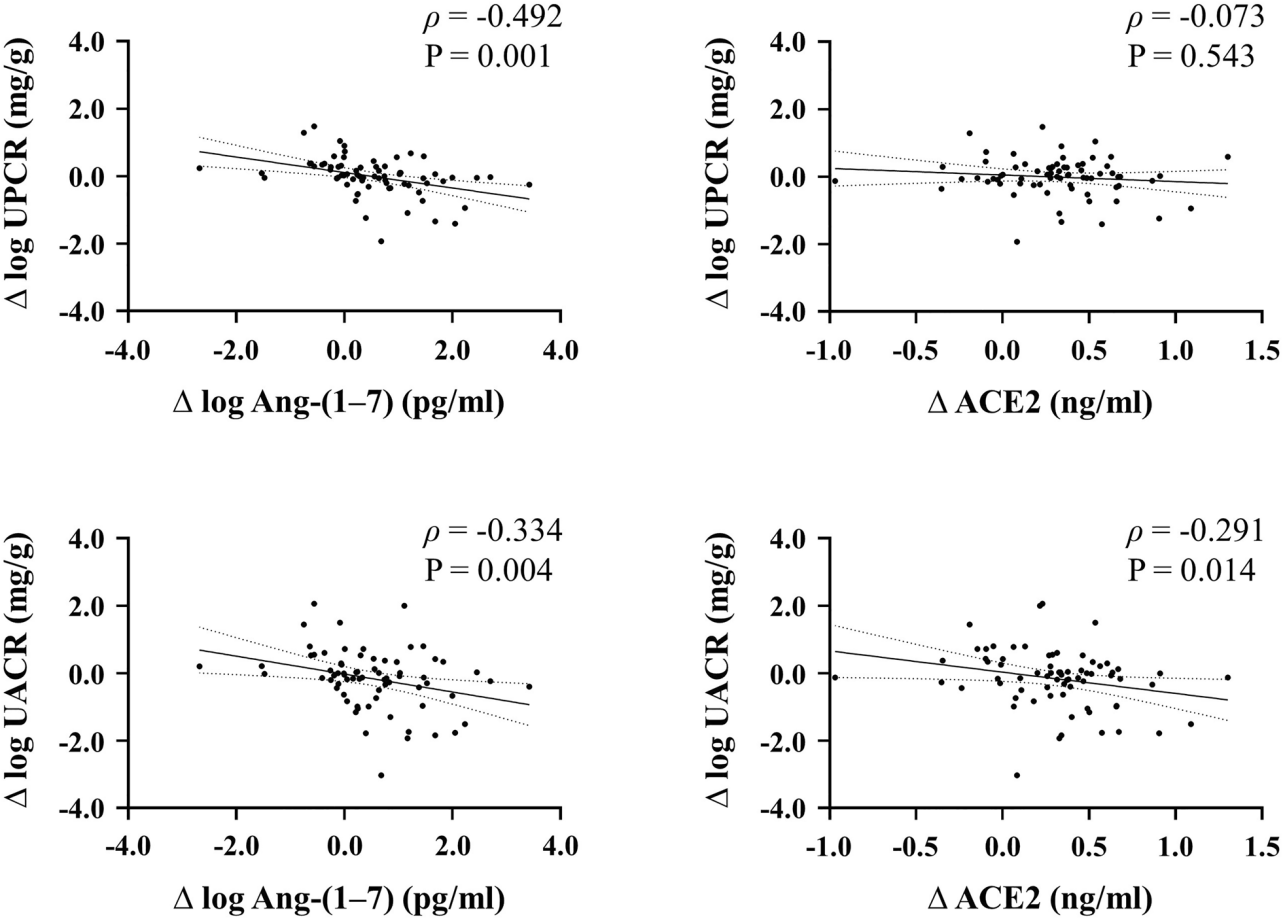
Correlation between Δ log Ang-(1–7) and Δlog UPCR or Δlog UACR, and between ΔACE2 and Δlog UPCR or Δlog UACR. *ACE2* angiotensin-converting enzyme 2, *Ang-(1–7)* angiotensin-(1–7), *UACR* urinary albumin-to-creatinine ratio, *UPCR* urinary protein-to-creatinine ratio; *∆* change (value at week 24—value at baseline)

In the correlation analysis using all participants, change in serum Ang-(1–7) levels was negatively associated with changes in UPCR (*r* =  − 0.394, P = 0.001) and UACR (*r* =  − 0.299, P = 0.011). Change in serum ACE2 levels was also negatively associated with a change in UACR (*r* =  − 0.252, P = 0.034; Fig. 3). In vascular function assessment, change in serum Ang-(1–7) levels was positively associated with an increase in PORH change (*r* = 0.241, P = 0.043) but not with an increase in FMD change. Change in serum ACE2 levels was not associated with an increase in FMD change or PORH change (Additional file 1: Fig. S2).

In simple regression analyses, changes in UPCR were positively correlated with the changes in HOMA-IR and aldosterone levels but negatively correlated with the changes in fasting glucose and Ang-(1–7). Changes in UACR were positively correlated with the changes in aldosterone levels but negatively correlated with the changes in SBP, DBP, fasting glucose, HbA1c, Ang-(1–7), and ACE2 levels. In multivariate regression analyses, changes in HOMA-IR, Ang-(1–7), and aldosterone levels were the significant determinants of the changes in UPCR (all *P* < 0.05) (Table 3). In addition, changes in Ang-(1–7), fasting glucose, PRA, and aldosterone were the significant determinants of the changes in UACR (all P < 0.05).

**Table 3:** Univariate and multivariate regression analyses for changes in UPCR and UACR

Univariate			Multivariate	
Variable	Coefficient	P	Standardized coefficient	P
∆ log UPCR				
Age	0.004	0.478	–	–
∆ Systolic BP	− 0.007	0.129	–	–
∆ Diastolic BP	− 0.003	0.525	–	–
∆ Fasting glucose	− 0.003	0.021	–	–
∆ HbA1c	− 0.092	0.232	–	–
∆ log HOMA-IR	0.333	0.005	0.258	0.010
∆ eGFR	0.003	0.427	–	–
∆ log hsCRP	0.112	0.199	–	–
∆ log Ang-(1–7)	− 0.229	0.001	− 0.350	0.001
∆ ACE2	0.000	0.320	–	–
∆ log PRA	− 0.042	0.480	–	–
∆ Aldosterone	0.033	< 0.001	0.406	< 0.001
			Model *R*^2^ = 0.400	
∆ log UACR				
Age	0.001	0.988	–	–
∆ Systolic BP	− 0.019	0.004	–	–
∆ Diastolic BP	− 0.023	0.005	–	–
∆ Fasting glucose	− 0.006	0.004	− 0.248	0.015
∆ HbA1c	− 0.232	0.045	–	–
∆ log HOMA-IR	0.357	0.053	–	–
∆ eGFR	− 0.001	0.911	–	–
∆ log hsCRP	0.158	0.223	–	–
∆ log Ang-(1–7)	− 0.265	0.011	− 0.202	0.040
∆ ACE2	− 0.001	0.034	–	–
∆ log PRA	− 0.152	0.090	− 0.265	0.008
∆ Aldosterone	0.052	< 0.001	0.452	< 0.001
			Model *R*^2^ = 0.426	

*UPCR* urinary protein-to-creatinine ratio, *UACR* urinary albumin-to-creatinine ratio, *HOMA-IR* homeostasis model assessment of insulin resistance; *eGFR* estimated glomerular filtration rate, *hsCRP* high-sensitivity C-reactive protein, *ACE2* angiotensin-converting enzyme-2, *PRA* plasma renin activity; *R*^*2*^ multiple coefficient of determination, *∆* change (the value at week 24 minus the value at baseline)

Treatments with both olmesartan and amlodipine were generally well tolerated. The incidence of adverse events was not different between groups (5.0% vs. 5.0%). No serious adverse events were observed in both groups. One case of dizziness and one case of hypotension were reported in the olmesartan group. One case of dizziness and one case of pruritus were reported in the amlodipine group (Additional file 1: Table S1).

## Discussion

In this randomized controlled trial, both olmesartan and amlodipine treatment for 24 weeks decreased SBP and DBP significantly, without any between-group differences. However, olmesartan treatment reduced urinary protein- or albumin-excretion rates, but amlodipine did not, leading to significant differences between the two groups. Notably, Ang-(1–7) and ACE2 levels increased more in the olmesartan group than in the amlodipine group. The increases in Ang-(1–7) and ACE2 levels were significantly correlated with the decrease in albuminuria. Based on the multivariate regression models, the increase in Ang-(1–7) levels was associated with a reduction in proteinuria and albuminuria. The increase in Ang-(1–7) levels was also associated with improvement in microcirculation measured using PORH change. These findings support the significant beneficial effects of olmesartan on the “nonclassical” RAS pathways that include Ang-(1–7) and ACE2.

It has been suggested that “nonclassical” RAS pathways, such as those involving ACE2, its product Ang-(1–7), and Mas receptor, might play a protective role in the cardiovascular continuum [5]. The cardiovascular and renal systems are the major sources of Ang-(1–7) production [25]. Ang-(1–7) and ACE2 have multifaceted effects on the heart and kidney, including vasodilatation, positive inotropic effects, myocardial protection, and inhibition of unfavorable cardiac remodeling and inflammation and fibrosis in kidneys [26, 27]. However, the favorable effect of Ang-(1–7) on the heart and blood vessels was only demonstrated in a preclinical study [5].

Regarding the effects of RAS blockade on Ang-(1–7) levels, previous studies showed that the ACE inhibitor captopril increased Ang-(1–7) levels in the tissue and plasma in rodents [28, 29] and plasma in humans [30]. Subcutaneous captopril treatment (5 mg/kg per 24 h) for 72 h significantly increased brain Ang-(1–7) levels in rats with focal cerebral ischemia [28]. In addition, oral captopril treatment (4.2 mg/kg) for 7 days significantly increased plasma Ang-(1–7) levels in rats [29]. In a human study, captopril treatment for 6 months significantly increased plasma Ang-(1–7) levels in participants with essential hypertension [30]. However, the effects of ARB on Ang-(1–7) level has not been investigated either in human or animal models. Our study is the first study that showed that an ARB, olmesartan, significantly increased plasma level of Ang-(1–7) in patients with diabetes and hypertension.

Few studies investigated the changes in ACE2 levels by treatment with RAS blocking agents. A hypertensive mouse model study showed that ACE inhibitor or ARB treatments increased ACE2 levels in tissue samples [13]. In a study with patients with type 1 diabetes, ACE inhibitor treatment increased serum ACE2 levels [31]. In contrast, another human study reported that circulating ACE2 levels were not changed by ACE inhibitor or ARB treatments [32]. This inconsistent finding might be explained by different study populations, such as those with heart failure [33], coronary artery disease [34], and myocardial infarction [35].

The relationship between serum Ang-(1–7) or ACE2 and albuminuria has not yet been studied in humans. A 2-week administration of Ang-(1–7) in hypertensive rats decreased proteinuria [36], while Ang-(1–7) receptor Mas knockout mice showed glomerular hyperfiltration and albuminuria [37]. The administration of recombinant human ACE2 attenuated albuminuria in a mouse model of diabetic nephropathy [38]. In addition, candesartan treatment for 4 weeks ameliorated albuminuria in a *db/db* mouse model, which was associated with increased plasma ACE2 levels [39]. In the present study, the increases in serum Ang-(1–7) and ACE2 levels as a result of olmesartan treatment were associated with a reduction in albuminuria, which is in accordance with the results of previous human and animal studies [14, 15]. In addition, multivariate regression analyses showed that increases in serum Ang-(1–7) levels were an independent predictor of a reduction in albuminuria.

Moreover, ACE2 levels might be related to an increased risk of morbidity and mortality in COVID-19. It was reported that severe acute respiratory syndrome coronavirus 2 binds to ACE2 and is internalized. This in turn leads to a downregulation of ACE2, which subsequently promotes AngII production [40], leading to an increased risk of CVDs [41]. ACE2 levels might also be involved in the association between COVID-19 and diabetes mellitus [17]. For example, ACE2-knockout mice were vulnerable to high-fat diet-induced pancreatic β-cell dysfunction [42]. Based on this finding, the increase in ACE2 levels by some RAS blocking agents, such as olmesartan, might help prevent or mitigate the development of CVDs in patients with COVID-19.

In the present study, endothelial function was measured using the FMD method and microcirculation was evaluated using PORH. These surrogate indices for vascular health were not significantly altered by olmesartan treatment. This may be explained by a relatively short period of treatment (24 weeks). It is noteworthy that the increase in Ang-(1–7) levels was positively correlated with improvement in PORH, possibly indicating the involvement of Ang-(1–7) in vascular health.

Previous studies have suggested beneficial effects of ARBs on metabolism and inflammation [43, 44]. On the contrary, our findings showed no significant changes in metabolic and inflammatory parameters after olmesartan treatment. LDL-cholesterol levels decreased in the olmesartan group but not in the amlodipine group, thereby leading to no between-group difference. Postprandial glucose levels were decreased in the olmesartan group while they were increased in the amlodipine group, resulting in a borderline significant between-group difference.

The overall incidence of adverse events was similar in both groups. The most commonly reported adverse effects of amlodipine and olmesartan were peripheral edema [45] and dizziness [46], respectively. In the present study, peripheral edema was not reported in both groups. However, one participant in the olmesartan group reported dizziness.

The present study has several strengths. To our knowledge, this study was the first randomized controlled trial study that investigated the effect of an ARB (olmesartan) and a CCB (amlodipine) on serum levels of Ang-(1–7) and ACE2. Albuminuria and vascular functions were also evaluated before and after treatment.

## Conclusion

Olmesartan treatment showed a significantly greater increase in the serum Ang-(1–7) and ACE2 levels, compared with amlodipine treatment. The increases in these novel RAS pathway parameters were correlated with a reduction in urinary albumin excretion in patients with T2D and hypertension. These findings suggest that Ang-(1–7) and ACE2 can be used as therapeutic targets for the prevention and treatment of diabetic kidney disease. Future definitive studies are needed to verify whether the effects of olmesartan on these novel RAS parameters would mediate renoprotective benefits.

## Supplementary Information

**Additional file 1: Fig. S1.** Study flow-chart. **Fig. S2.** Correlation between Δ log Ang-(1–7) and Δ FMD change or Δ PORH change, and between Δ ACE2 and Δ FMD change or Δ PORH change. *ACE2* angiotensin-converting enzyme 2, *Ang-(1–7)* angiotensin (1-7); *FMD* flow-mediated vasodilatation; *PORH* post-occlusive reactive hyperemia, *∆* change (value at week 24— value at baseline). **Table S1.** Number of patients with adverse events.

## Data Availability

The datasets generated during the current study are available from the corresponding author on reasonable request.

## Abbreviations

RAS: Renin-angiotensin system
CVD: Cardiovascular diseases
AngII: Angiotensin II
ACE: Angiotensin-converting enzyme
Ang-(1–7): Angiotensin-(1–7)
ARB: Angiotensin II receptor blocker
T2D: Type 2 diabetes
COVID-19: Coronavirus disease 2019
SBP: Systolic blood pressure
DBP: Diastolic blood pressure
CCB: Calcium channel blocker
PRA: Plasma renin activity
FMD: Flow-mediated vasodilatation
FPG: Fasting plasma glucose
HDL: High-density lipoprotein
LDL: Low-density lipoprotein
hsCRP: High-sensitivity C-reactive protein
eGFR: Estimated glomerular filtration rate
