# Supplementary material for: Effect of olmesartan and amlodipine on serum angiotensin-(1–7) levels and kidney and vascular function in patients with type 2 diabetes and hypertension
Source: Diabetol Metab Syndr. 2023 Mar 11;15:43. doi: 10.1186/s13098-023-00987-1 (PMC10005920; doi:10.1186/s13098-023-00987-1)
Supplement: Supplementary file 1 — Additional file 1: Fig. S1. Study flow-chart. Fig. S2. Correlation between Δ log Ang-(1–7) and Δ FMD change or Δ PORH change, and between Δ ACE2 and Δ FMD change or Δ PORH change. ACE2 angiotensin-converting enzyme 2, Ang-(1–7) angiotensin (1-7); FMD flow-mediated vasodilatation; PORH post-occlusive reactive hyperemia, ∆ change (value at week 24— value at baseline). Table S1. Number of patients with adverse events. [file 13098_2023_987_MOESM1_ESM.docx]

**Additional file 1: Fig. S1** Study flow-chart.


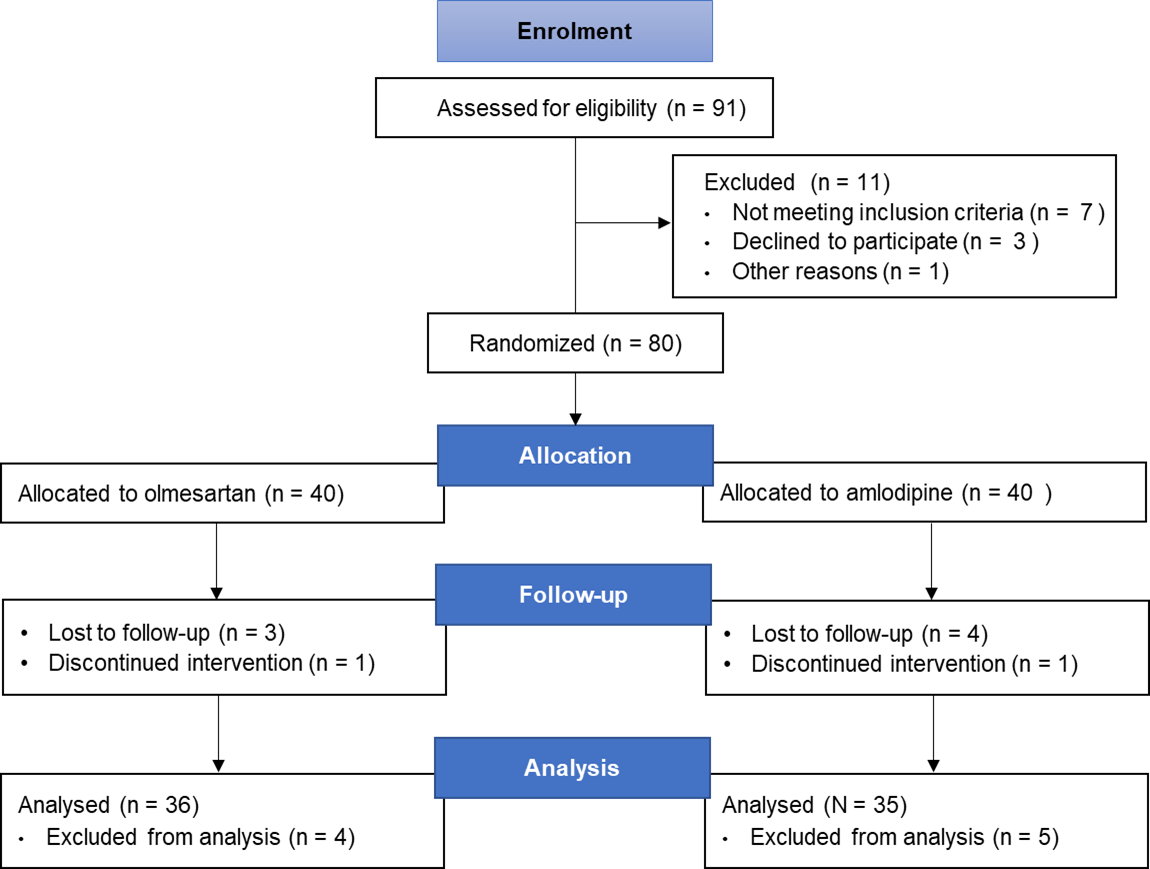


**Additional file 1: Fig. S2** Correlation between Δ log Ang-(1–7) and Δ FMD change or Δ PORH change, and between Δ ACE2 and Δ FMD change or Δ PORH change. ACE2, angiotensin-converting enzyme 2; Ang-(1–7), angiotensin (1-7); FMD, flow-mediated vasodilatation; PORH, post-occlusive reactive hyperemia; ∆, change (value at week 24 – value at baseline).


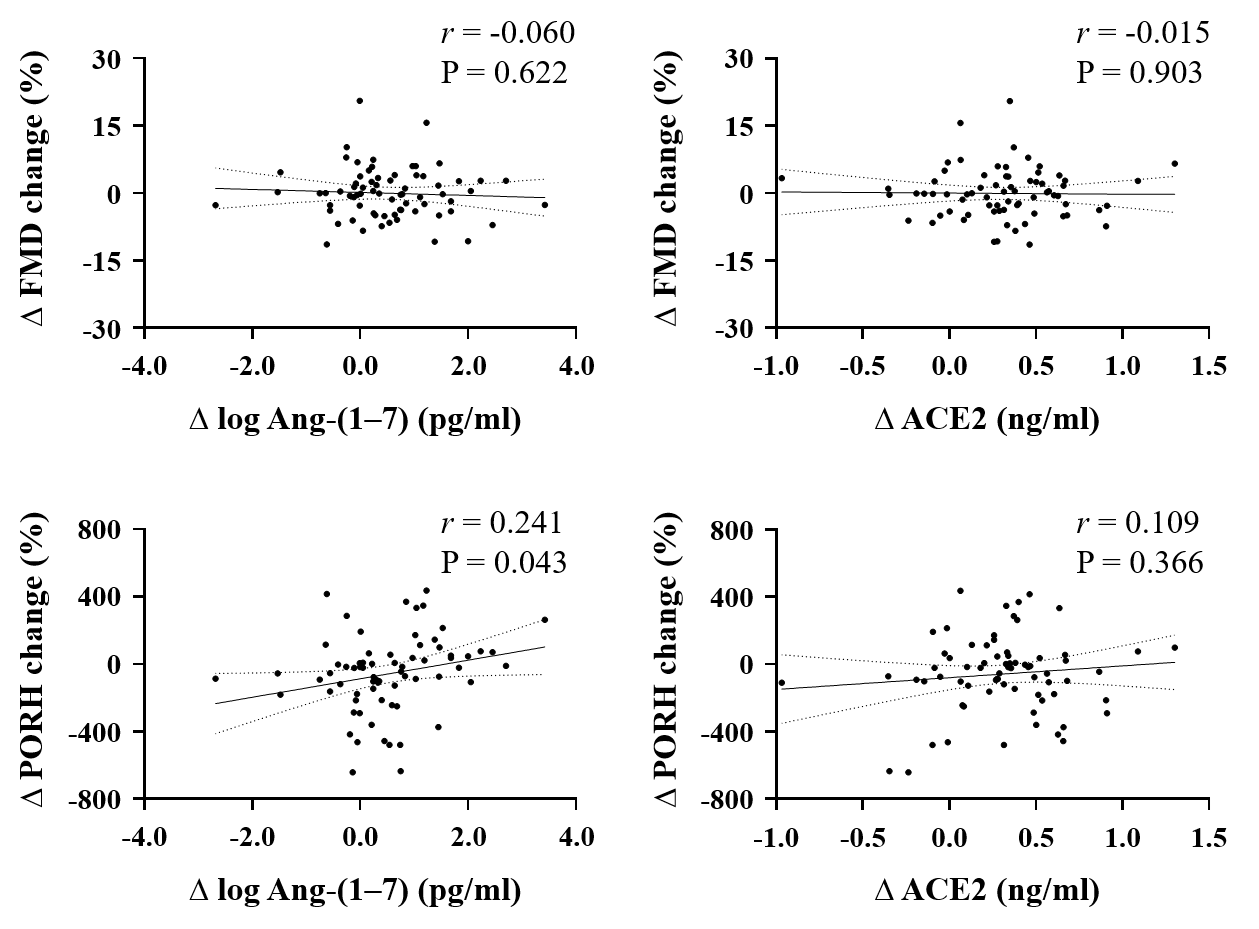


**Additional file 1: Table S1**

| **Table S1 Number of patients with adverse events** | | |
| --- | --- | --- |
|  | Olmesartan (N = 40) | Amlodipine (N = 40) |
| Total adverse events, N (%) | 2 (5.0) | 2 (5.0) |
| Adverse events leading to discontinuation, N (%) | 0 (0.0) | 0 (0.0) |
| Serious adverse events, N (%) | 0 (0.0) | 0 (0.0) |
| Any adverse event, N (%) |  |  |
| Headache | 0 (0.0) | 0 (0.0) |
| Nasopharyngitis | 0 (0.0) | 0 (0.0) |
| Back pain | 0 (0.0) | 0 (0.0) |
| Dizziness | 1 (2.5) | 1 (2.5) |
| Peripheral edema | 0 (0.0) | 0 (0.0) |
| Pruritus | 0 (0.0) | 1 (2.5) |
| Hypotension | 1 (2.5) | 0 (0.0) |
| Hyperkalemia | 0 (0.0) | 0 (0.0) |
| Data represent the number of patients (%). | | |
